# Gender differences in time to first hospital admission at age 60 in Denmark, 1995–2014

**DOI:** 10.1007/s10433-021-00614-w

**Published:** 2021-03-27

**Authors:** Andreas Höhn, Anna Oksuzyan, Rune Lindahl-Jacobsen, Kaare Christensen, Rosie Seaman

**Affiliations:** 1 Institute of Genetics and Molecular Medicine, University of Edinburgh, Edinburgh, UK; 2 Max Planck Institute for Demographic Research, Rostock, Germany; 3 Department of Epidemiology, Biostatistics, and Biodemography, University of Southern Denmark, Odense, Denmark; 4 Interdisciplinary Centre On Population Dynamics (CPop), University of Southern Denmark, Odense, Denmark; 5 Danish Ageing Research Centre, University of Southern Denmark, Odense, Denmark; 6 University of Stirling, Stirling, UK

**Keywords:** Gender differences, Hospital admissions, Healthcare use, Health

## Abstract

Women have consistently lower mortality rates than men at all ages and with respect to most causes. However, gender differences regarding hospital admission rates are more mixed, varying across ages and causes. A number of intuitive metrics have previously been used to explore changes in hospital admissions over time, but have not explicitly quantified the gender gap or estimated the cumulative contribution from cause-specific admission rates. Using register data for the total Danish population between 1995 and 2014, we estimated the time to first hospital admission for Danish men and women aged 60. This is an intuitive population-level metric with the same interpretive and mathematical properties as period life expectancy. Using a decomposition approach, we were able to quantify the cumulative contributions from eight causes of hospital admission to the gender gap in time to first hospital admission. Between 1995 and 2014, time to first admission increased for both, men (7.6 to 9.4 years) and women (8.3 to 10.3 years). However, the magnitude of gender differences in time to first admission remained relatively stable within this time period (0.7 years in 1995, 0.9 years in 2014). After age 60, Danish men had consistently higher rates of admission for cardiovascular conditions and neoplasms, but lower rates of admission for injuries, musculoskeletal disorders, and sex-specific causes. Although admission rates for both genders have generally declined over the last decades, the same major causes of admission accounted for the gender gap. Persistent gender differences in causes of admission are, therefore, important to consider when planning the delivery of health care in times of population ageing.

**Supplementary Information:**

The online version contains supplementary material available at 10.1007/s10433-021-00614-w.

## Introduction

On average, women live longer than men in all countries of the world. The gender gap in life expectancy has persisted over time, but it has changed in magnitude, from increasing throughout the twentieth century to decreasing thereafter (Thorslund et al. 2013). The contribution different causes of death have made to the narrowing gender gap in life expectancy are relatively well established. For example, attention has been drawn to the relatively faster declines in deaths from circulatory and smoking-related diseases among men throughout the end of the twentieth century (Sundberg et al. 2018). Despite these improvements, the most recent estimates for countries within the European Union show that the gender gap in life expectancy is between 3.2 years and 9.9 year (EuroStat. 2018).

Gender differences in life expectancy have important implications for how healthcare responds to population ageing. Hospital admissions are a fundamental element of the healthcare system which may be particularly vulnerable to population ageing and where gender differences are evident. Before the age of 60, women are more likely to be admitted to hospital than men due to obstetrics-related admissions (Westergaard et al. 2019). After the age of 60 and as the incidence of major non-communicable diseases increases, women are less likely to be admitted to hospital than men (Westergaard et al. 2019). Examining gender differences by cause of admission to hospital is common practice in health research, with men tending to have higher all-cause admission rates, as well as higher rates of admission for the most acute and life threatening conditions (Case and Paxson 2005). In contrast, women tend to have a higher risk of admission to hospital for less severe but often disabling conditions (Luben et al. 2016; Simmonds et al. 2014).

A number of intuitive metrics have previously been used to explore changes in hospital admissions over time. These include the average age of patients being treated in hospitals (NHS Digital (2016)), the average age at first hospital diagnosis (Modig et al. 2019; Westergaard et al. 2019), and whether increases in the average age at first admission have kept pace with increases in life expectancy (Karampampa et al. 2013). However, these studies did not explicitly set out to examine the gender gap in hospital admissions. Using individual-level register data for the total Danish population, we estimated time to first hospital admission after age 60 for men and women of exact age 60, between 1995 and 2014. We introduce time to first hospital admission after age 60 as an intuitive, population-level metric which has the same interpretative and mathematical properties as remaining life expectancy at age 60. Our measure, therefore, comes with the benefit that it can be decomposed into the cumulative contributions from different causes of hospital admission to identify how gender differences in health have changed over time.

In line with trends for life expectancy, we expected time to first admission to increase among Danish men and women and gender differences in time to first admission to decrease. Furthermore, we expected cardiovascular diseases and neoplasms to account for the declines in the magnitude of gender differences in time to first hospital admission.

## Methods and materials

### Data sources

In this study, we used individual-level register data covering the total Danish population. Using the unique personal identification number (CPR-Number), we linked data from the Central Population Register (CPR) with records from the National Patient Register (NPR). The CPR covers general socio-demographic information on the population alive and residing in Denmark since 1968, including data on gender and the date of birth (Schmidt et al. 2014). The NPR, a population-based register with high levels of completeness and reliability, contains information on all treatments provided in Danish hospitals since 1977, such as type, length and cause of admission to hospital (Lynge et al. 2011). In the NPR, information on outpatient treatments, admissions to psychiatric wards and psychiatric hospitals, and emergency admissions have only been recorded since 1995 (Schmidt et al. 2015). We chose to begin the study period in 1995, in order to investigate the sensitivity of our results to the inclusion and exclusion of outpatient treatments and emergency admissions. Denmark introduced the 10th Revision of the International Classification of Diseases (ICD-10) in 1994 (Munk-Jørgensen et al. 1999). We were, therefore, able to group causes of hospital admission using the main chapters of ICD-10 consistently throughout the entire study period.

### Study population

We did not follow one single study population continuously over calendar time. Instead, we identified and followed a synthetic cohort study population within each calendar year of the study period (Seaman et al. 2020). In the first step, we identified all individuals aged 60 and older alive and residing in Denmark on 1 January of a particular year (for example: 2014). In the second step, we identified the population at risk of a first admission to hospital in that particular year. In order to identify this population at risk, we identified and excluded all individuals from the study population, who were hospitalized within a previous 7-year period (for the example year 2014: 2007–2013) irrespective of the length of stay or the cause of admission to hospital. This is referred to as the 7-year washout period and was guided by existing literature (Modig et al. 2017, 2019). Applying a washout period aims to limit the chance that a first event is a readmission or a follow-up treatment (Karampampa et al. 2013). In the third step, we identified those individuals who had a first event within each calendar year. Our main results defined an event as the first inpatient admission to hospital with a minimum of 2 treatment days (equivalent to one overnight stay) and included cases that ended in either death or discharge. This definition is likely to have captured conditions of some severity at the time of admission.

**Fig. 1:**
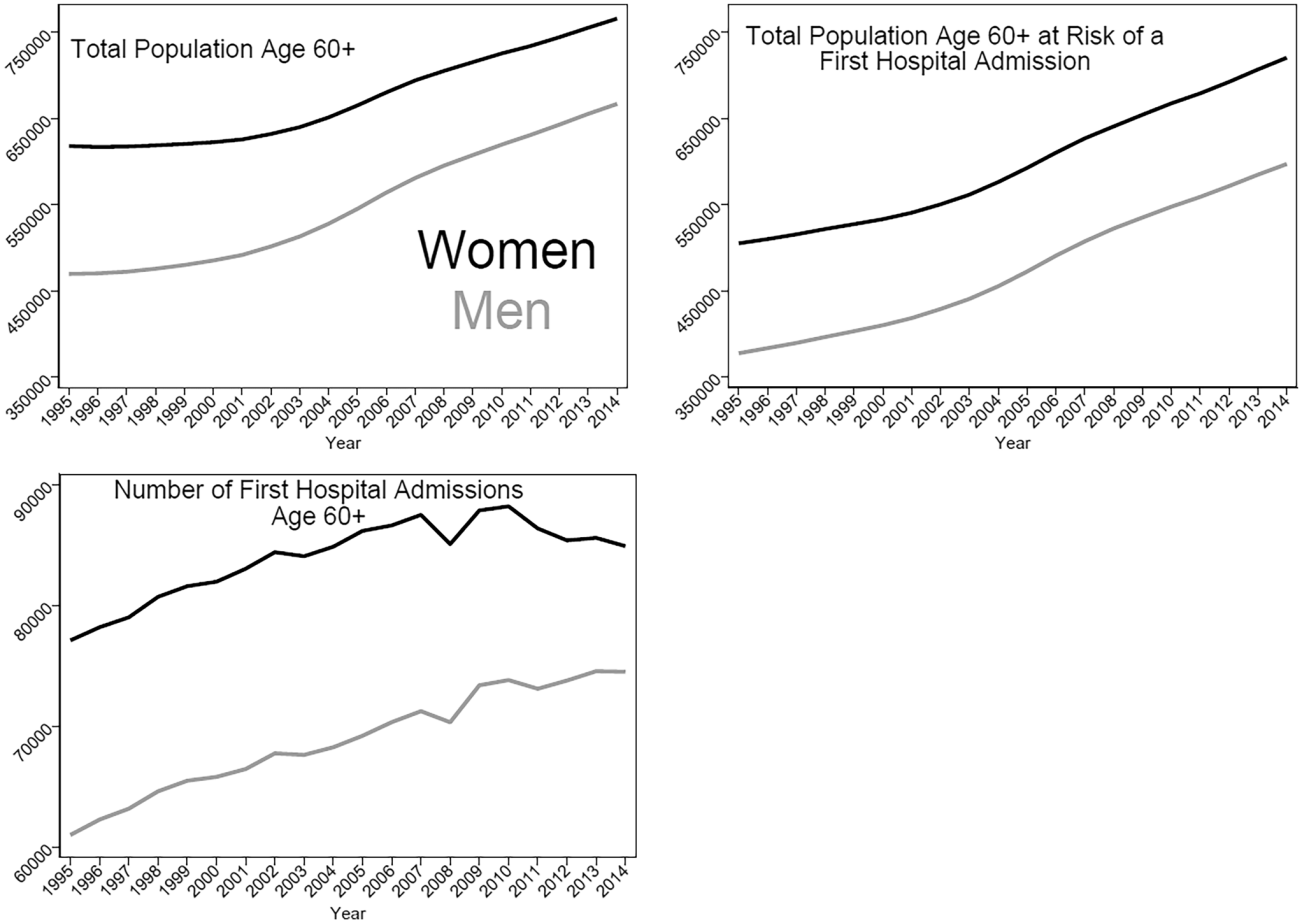
Number of all Danish Men and Women aged 60+, men and women aged 60+ at risk of first admission, and number of first hospital admissions among men and women aged 60+, 1995 to 2014

Figure 1 provides a summarizing overview of the number of Danish Men and Women aged 60 and older, Danish men and women aged 60 and older defined as at risk of first admission, and the number of defined first hospital admissions among Danish men and women aged 60 and older for each calendar year between 1995 and 2014.

### Methods

In a first step, we estimated the time to first all-cause hospital admission for Danish men and women of exact age 60 using the standard life table methodology, which is commonly used to derive period life tables for mortality (Chiang 1984). With our application of the life table methodology to hospital admission data, we followed an already existing approach (Seaman et al. 2020), but extend this approach by accounting for the fact that the all-cause risk of an event—first hospital admission—can be split into the contributing fractions—causes of admission (Preston et al. 2001). We divided the number of first events by the population at risk in order to estimate the age-specific risks of a first all-cause admission to hospital ($q_{x,t} hosp$), separately for men and women, by single years of age *x*, and for each calendar year *t*. This age-specific, all-cause risk of hospitalization is the sum over all studied causes of admissions to hospital $i$—($q_{x,t,i} hosp$). Our definition of $e_{x,t} hosp$ is similar to the definition of remaining life expectancy at age *x* in year $t$ ($e_{x,t}$) in a standard period life table using information on mortality. In our case, $e_{60,t} hosp$ quantifies the remaining average number of years until individuals of exact age 60 experience their first admission to hospital after the age of 60, conditional upon survival to age 60, and given hospitalisation patterns of year *t*.

In a second step, we examined the causes accounting for the gender gap in time to first hospital admission after age 60 at different points in time. For this purpose, a range of decomposition methods were available. All decomposition are based on the assumption that the difference in two population-level measures—for example, the difference in life expectancy between men and women—can be split into the contribution of different covariates—for example, the contribution of various causes of death (Ponnapalli 2005). To disentangle the impact of different causes of admission on the gender gap in time to first hospital admission, we decided to use Horiuchi et al. (2008) decomposition method. This method follows the well-established and very similar approaches of Andreev, Pollard, and Arriaga closely (Ponnapalli 2005), but assumes the impact of covariates to change continuously rather than discretely (Horiuchi et al. 2008). We decomposed the gender gap in $e_{60,t} hosp$ at the start (1995), the mid-point (2005), and the end (2014) of the study period.

We distinguished between eight major causes of admission, derived from the main ICD-10 chapters: respiratory disease, neoplasms, circulatory diseases, digestive diseases, musculoskeletal disorders, injuries and poisonings, sex-specific causes, and other causes of admission. Major categories of causes of admission were chosen to decrease the potential for misclassification (Lahti and Penttila 2001). The ICD-10 codes assigned to each category are outlined in Supplementary Material 1. We chose to identify sex-specific causes of admission as an independent cause of admission category. This approach was chosen to make the other major categories (e.g., neoplasms) reflect causes of admission that both men and women could be admitted to hospital for. While we recognize that sex-specific causes are not directly comparable between men and women, there may be parallels to be drawn (Sundberg et al. 2018).

Merging of registries and data preparation were carried out with *Stata* (Version 14). All analyses were carried out using the *DemoDecomp* Package (Riffe 2018) for *R* (Version 3.6).

## Results

### Time to first hospital admission

**Fig. 2:**
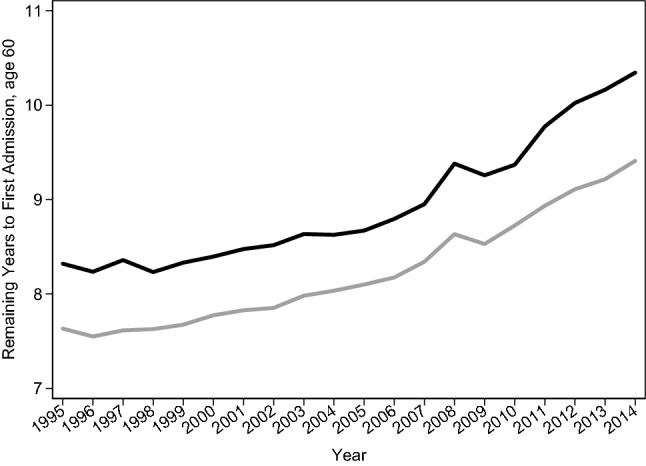
Trends in remaining time to first hospital admission for Danish men and women of exact age 60, for inpatient admissions lasting for a minimum of 2 treatment days, 1995 to 2014. Note: The peak between 2007 and 2009 corresponds with administrative restructuring of healthcare in Denmark and the centralization of hospital care into fewer hospitals (Christiansen and Vrangbaek 2018)

Figure 2 shows the trend in time to first admission for Danish men and women of exact age 60 for inpatient stays lasting ≥ 2 days. We focussed on the start (1995), the mid-point (2005), and the end of the study period (2014). Our results show that women consistently experienced a longer time to first admission, suggesting that 60-year old women can expect to live for longer without being admitted to hospital than men. Time to first hospital admission after age 60 increased steadily for both men and women over the study period. This indicates that individuals experienced their first admission to hospital at later ages in 2014. In 1995, time to first admission was 7.6 years for men and 8.3 years for women. By 2005, the levels increased to 8.1 years among men (+ 0.5 years) and 8.7 years among women (+ 0.4 years). Between 2005 and 2014, time to first hospital admission increased more rapidly and reached 9.4 years among men (+ 1.3 years) and 10.3 years among women (+ 1.7 years). The gender gap in remaining time to first admission was relatively stable within this period and changed only slightly from 0.7 years in 1995, to 0.6 years in 2005, and 0.9 years in 2014.

### Decomposing the gender gap

**Fig. 3:**
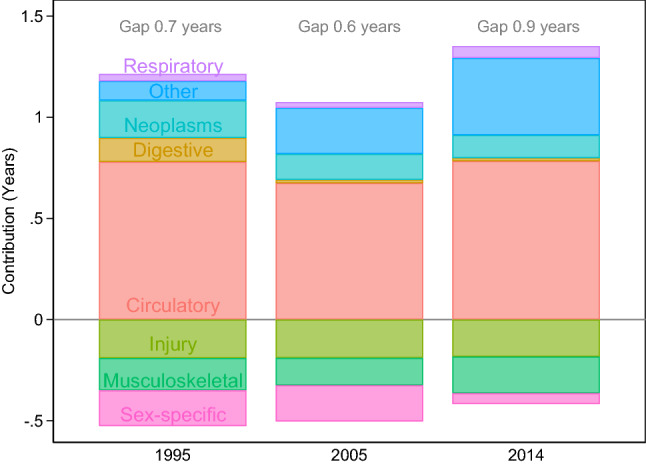
Cause-specific contributions to the gender gap in remaining time to first admission after age 60 for inpatient admissions lasting for a minimum of 2 treatment days, 1995 and 2014. Note: neoplasms: excluding sex-specific neoplasms

Figure 3 shows the contributions of eight different causes of admission to the gender gap in time to first hospital admission after age 60 in 1995, 2005, and 2014. The sum over all contributions is the total gender gap in time to first admission within each of the three years—the value of which is stated at the top of the relevant bar. The larger the contribution, the cause of hospital admission makes the greater and the gender difference among the underlying cause-specific hospital admission rates. For example, contributions above the zero line reflect causes where men had higher underlying rates of admission than women. Contributions below the zero line reflect causes where women had higher underlying rates of admission than men.

Figure 3 shows that similar cause-specific contributions accounted for the gender gap in 1995, 2005, and 2014. The largest contributing single cause of admission to the gender gap was circulatory diseases. Circulatory diseases contributed 0.8 years to the total gender gap in 1995, 0.7 years in 2005, and 0.8 years in 2014. Men also experienced higher admission rates for digestive diseases, neoplasms, respiratory diseases, and all other causes of admission. The contributions from respiratory diseases to the gender gap in remaining time to first admission after age 60 were > 0.1 years at all three time points. The contribution from neoplasms (1995: 0.2 years; 2005: 0.1 years; 2014: 0.1 years) and from digestive diseases (1995: 0.1 years; 2005: < 0.1 years; 2014: < 0.1 years) to the gender gap decreased during the study period. The contribution from other causes increased substantially within the study period, accounting for the largest absolute change: from 0.1 in 1995, to 0.2 in 2005, and 0.4 in 2014.

Figure 3 highlights that, after the age of 60, men did not have higher admission rates for all causes of admission. For example, men experienced lower admission rates than women for injuries, musculoskeletal disorders, and sex-specific causes of admission. The contribution of musculoskeletal disorders (1995: −0.1; 2005:−0.2; 2014: −0.1), as well as the contribution from injuries (1995: −0.2; 2005:−0.2; 2014: −0.2), changed only slightly within the study period. In contrast to this, the contribution from sex-specific causes decreased significantly (1995: −0.2; 2005: -0.2; 2014: < -−0.1). To gain a deeper understanding of the changing contribution from sex-specific causes, we analysed the more detailed diagnostic codes (see Supplementary Material 2).

### Sensitivity analysis

Our main results defined an admission to hospital as an inpatient stay lasting a minimum of 2 treatment days. Since 1995, it has been possible to include different types of hospital admissions such as outpatient treatments and emergency admissions (Schmidt et al. 2015). Therefore, we repeated our main analysis, which included inpatient admissions only, for all types of hospital admissions together which lasted a minimum of 2 treatment days. We decided to keep the criterion of 2 or more treatment days, in order to ensure that changes in our findings are attributable to changes in the type of admission only—and not the result of a combination of type and duration.

When including all types of hospital admission, remaining time to first hospital admission after age 60 was lower for both men and women, compared with remaining time to first hospital admission for inpatient stays only. The trend line for all hospital admissions was decreasing initially but returned increasing following 2010. The gender gap was much smaller when looking at all types of admission. However, the cause-specific contributions to the gender gap were very similar to those found when analysing inpatient admissions only.

**Fig. 4:**
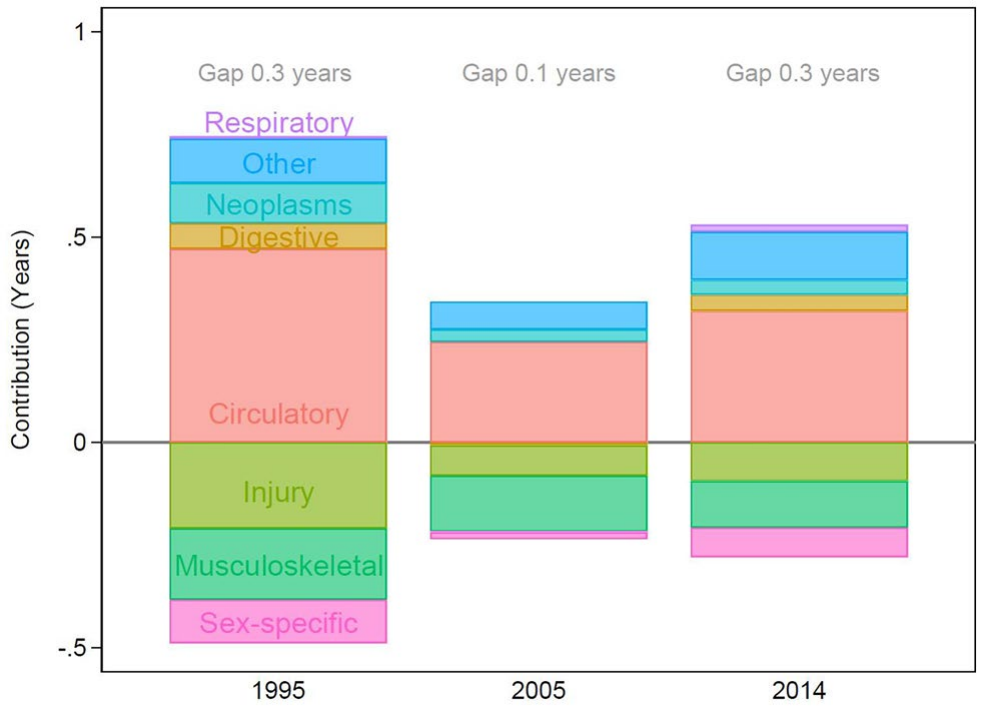
Cause-specific contributions to the gender gap in remaining years to first admission after age 60 for inpatient, outpatient and emergency admissions lasting for a minimum of 2 treatment days, 1995 and 2014. Note: neoplasms: without explicitly sex-specific neoplasms

Figure 4 shows the decomposition results including all types of hospital admission lasting a minimum of 2 treatment days. Gender differences in the cause-specific contributions remained similar to those found when only looking at inpatient admissions: Women had a disadvantage for injuries, musculoskeletal, and sex-specific causes of admission. Men had a disadvantage for circulatory diseases, digestive disease, neoplasms, respiratory diseases, and all other causes of admission (for more details: see Supplementary Material 3).

## Discussion

### Summary of main findings

Defining hospital stays as inpatient stays lasting  ≥ 2 days, we found that time to first hospital admission after age 60 increased among Danish men and women between 1995 and 2014. Within this period, the gender gap in time to first admission remained relatively stable. Throughout the study period, circulatory diseases were the largest single cause of admission accounting for the gender gap. Results of sensitivity analyses, defining hospital stays as any type of hospital admission lasting ≥ 2 days, showed a decrease in time to first admission. For this definition of hospital stay, we found a smaller gender gap in remaining time to first admission, although the underlying differences in cause-specific contributions were very similar.

### Interpretations

In most developed countries, the gender gap in life expectancy has narrowed over the last 50 years. This is largely attributable to decreasing differences in mortality rates from circulatory diseases and neoplasms between men and women (Sundberg et al. 2018; Thorslund et al. 2013). Converging mortality rates between men and women point towards the fact that health and health behaviours among men and women may have become more similar (Gjonca et al. 2005; Glei and Horiuchi 2007). We anticipated increases in time to admission for both men and women alongside a narrowing gender gap. While time to first admission increased for both men and women, the gender gap widened slightly.

One potential explanation for the increase in time to first admission among men and women could be that individuals are ageing healthier. Supporting this assumption, it has been shown that the age at onset of major cardiovascular events has shifted towards older ages (Modig et al. 2019). In addition, falling smoking rates and the reduction in smoking-related conditions, including respiratory conditions, neoplasms and most cardiovascular conditions (Demant et al. 2013; Schmidt et al. 2012; Thun et al. 2012), might have contributed to the increase in time to first admission. Further evidence for healthy ageing has been reported for a number of health dimensions, including physical health and cognitive functioning and have been attributed to improvements in medical technology, health behaviour, and early life conditions (Christensen et al. 2009).

An alternative interpretation is that increases in time to first admission could have emerged from changes to treatment and admission strategies. For example, conditions which were previously treated over multiple days within an inpatient setting could have been pushed towards outpatient departments or primary care (Galenkamp et al. 2016). We found that the contributions from most causes of admission to the gender gap were consistent across our sensitivity analysis comparing inpatient stays with all hospital stays. The category “other causes” was an exception. The main analyses, including only inpatient stays lasting ≥ 2 days, showed that “other causes” was the category with the largest increases in the contribution to the gender gap over time. In contrast, the sensitivity analysis including all hospital stays lasting ≥ 2 days suggested that the contribution of “other causes” did not change as much. Unfortunately, we were not able to account for any systematic changes to admission and treatment strategies. More detailed diagnostic information and data from primary care would be required to identify if systematic changes to admission or treatment strategies accounted for the contrasting findings for “other causes”. However, a more detailed analysis of causes of admission would have been subject to a larger misclassification bias, which typically increases with the level of diagnostic detail (O'Malley et al. 2005). We also found no evidence in the literature indicating that changes in admission and treatment strategies would have had a differential impact on men and women.

Our study indicates that the time to first admission after age 60 was consistently higher for women than for men. One underlying mechanism for gender differences in time to first admission may be gender differences in disease progression and health deterioration (Höhn et al. 2018). Evidence has shown that, after age 60, men experience higher all-cause and cause-specific hospital admissions rates than women (Jensen et al. 2014)—even when they have the same underlying health conditions, engage in similar life styles, and when controlling for socio-demographic characteristics (Case and Paxson 2005). This has been interpreted as evidence of more rapid disease progression among men.

Faster disease progression among men may be compounded by gender differences in treatment-seeking behaviour. In contrast to hospital admissions, women tend to have higher levels of primary healthcare use than men (Banks and Baker 2013; Wang et al. 2013). In the past, studies have often interpreted this finding as an indicator of a health advantage among men (Hunt et al. 2011). However, it has been argued that lower levels of primary healthcare use among men are more likely to reflect their reluctance to seek medical advice. This reluctance to seek advice early on might lead to delays in diagnosis and treatment, causing diseases to progress more rapidly to the point where treatment in hospital is necessary (Höhn et al. 2020).

Mirroring decreasing mortality differences between men and women from cardiovascular disease and neoplasms (Sundberg et al. 2018; Thorslund et al. 2013), we expected the gender gap in time to first hospital admission to decrease. However, we found gender differences in time to first hospital admission to be very persistent throughout the study period and to even widen slightly. It is possible that this overarching stable pattern could have emerged from two opposing trends. On the one hand side, sex differences in the incidence of cardiovascular conditions, such as myocardial infarction, decreased in Denmark within the studied period as improvements were larger among men than among women (Schmidt et al. 2012). On the other hand, the unique pattern of smoking among Danish women born between 1919 and 1939 (Lindahl-Jacobsen et al. 2016) and its fading lag effect could have contributed to a widening gender gap in time to first hospital admission. Smoking was one of the largest contributing factors to stagnating life expectancy among Danish women between 1977 and 1995 and is also important to consider in relation to our results. Our study population included individuals aged 60 and older. This means that women from the cohort born in 1919 would have been 76 years old in 1995, while women born in 1939 would have first entered the study population in 1998, when they turned 60. Smoking and its well-established lag effects on health have important implications for patterns observed among all-cause and cause-specific admission rates (Hanlon et al. 2007). If hospital admissions among women in Denmark were elevated due to the health behaviours of previous cohorts, then the increasing gender gap seems like a consequence as healthier cohorts of women enter the study population. This also means that the magnitude of the gender gap in time to first hospital admission may be smaller in Denmark than in other countries, where smoking rates among women remained low.

### Strengths and limitations

In Denmark, access to healthcare is free of charge and universal (Pedersen et al. 2012). This substantially minimizes selection into healthcare and allows individuals to utilize healthcare when needed (Olejaz et al. 2012). Therefore, our results may not be generalizable to healthcare systems where out-of-pocket payments are high. Additional consideration should be given to the particular role GPs in Denmark play for determining an admission to hospital. GPs are gatekeepers for the use of hospital care in Denmark, unless patients are admitted to hospital in an emergency or with an acute onset of conditions. It could also be possible that a GP’s decision to admit (or to not admit) a patient to hospital is impacted by the patient’s gender and that these gender-specific admission thresholds could have changed over time. Furthermore, our results may not be comparable to healthcare systems with a larger proportion of inpatient capacities and private hospitals or where the gate-keeping function of GPs differs from that in Denmark.

Our measure, time to first hospital admission, is subject to limitations. Individuals are likely to be admitted to hospital multiple times and for different causes. We identified the first admission to hospital after age 60 and applied a washout period. Applying a washout period aimed to reduce the chances that a first admission was a readmission or a follow-up treatment. However, this means that our definition of a first admission after age 60 might not always be the “true” first admission. For example, an individual aged 80 and admitted to hospital in 2010 might have experienced an admission at age 70 in 2000 for the same or a different condition.

Another limitation emerges from the use of a synthetic cohort study design—a perspective originating from demography and actuarial sciences (Modig et al. 2020). Analogously to the limitations of period life expectancy, our measure is based on the average of a temporary snapshot of all age groups combined in one single year (Modig et al. 2020). Changes in time to first hospital admission might be subject to period- and cohort effects which remained unexplained in our study. Using a synthetic cohort study design aimed to ensure comparability over time and to maximise the amount of data included in each year.

A major strength of this study is the high-quality Danish register data that was used. By linking the CPR with the NPR, we were able to follow the total Danish population for hospital admissions. These data substantially reduced the known biases that typically emerge when using survey data, such as loss to follow-up, recall-bias, non-response, and reporting styles (Thygesen and Ersbøll 2014). While findings where not always consistent, studies have shown that these aspects might differ over the life course and between men and women (Layes et al. 2012; Oksuzyan et al. 2019). This has important implications, for example, regarding the generalizability and interpretation of results from survey data surrounding the direction and magnitude of gender gaps in health and healthcare use.

## Conclusion

Previous studies using routinely-collected hospital records have often only been able to include recorded hospital admissions. We were able to capture the underlying population at risk to estimate a novel and intuitive population-level metric of health. Our measure, time to first hospital admission, has the same interpretation and mathematical properties as the well-established metric of period life expectancy. This meant that we were able to facilitate population-level comparisons over time and to decompose the cumulative contribution of different causes of admission to the gender gap in time to first admission. While gender differences in life expectancy have narrowed, gender differences in hospital admissions remained unchanged. Persistent gender differences in causes of admission are, therefore, important to consider when planning the delivery of health care in times of population ageing. A better understanding of the underlying mechanisms for gender differences in hospital admissions is needed to ensure that hospitals are prepared for the challenges of population ageing.

## Supplementary Information

Below is the link to the electronic supplementary material.

Supplementary file1 (DOCX 12 kb)

Supplementary file2 (DOCX 73 kb)

Supplementary file3 (DOCX 52 kb)

## References

[CR1] Banks I, Baker P (2013). Men and primary care: improving access and outcomes Trends in Urology & Men’s. Health.

[CR2] Case A, Paxson C (2005). Sex differences in morbidity and mortality. Demography.

[CR3] Chiang CL (1984). The life table and its applications.

[CR4] Christensen K, Doblhammer G, Rau R, Vaupel JW (2009). Ageing populations: the challenges ahead. The Lancet.

[CR5] Christiansen T, Vrangbaek K (2018). Hospital centralization and performance in Denmark-Ten years on. Health Policy.

[CR6] Demant MN (2013). Temporal trends in stroke admissions in Denmark 1997–2009. BMC Neurol.

[CR7] NHS Digital (2016) Hospital Admitted Patient Care Activity, 2015–2016

[CR8] EuroStat. (2018) Life expectancy at birth in the EU: men versus women. https://ec.europa.eu/eurostat/web/products-eurostat-news/-/DDN-20190725-1

[CR9] Galenkamp H, Deeg DJH, de Jongh RT, Kardaun JWPF, Huisman M (2016). Trend study on the association between hospital admissions and the health of Dutch older adults (1995–2009). BMJ Open.

[CR10] Gjonca A, Tomassini C, Toson B, Smallwood S (2005). Sex differences in mortality, a comparison of the United Kingdom and other developed countries. Health Stat Q.

[CR11] Glei DA, Horiuchi S (2007). The narrowing sex differential in life expectancy in high-income populations: effects of differences in the age pattern of mortality. Popul Stud (Camb).

[CR12] Hanlon P, Lawder R, Elders A, Clark D, Walsh D, Whyte B, Sutton M (2007). An analysis of the link between behavioural, biological and social risk factors and subsequent hospital admission in Scotland. J Public health.

[CR13] Höhn A, Larsen LA, Schneider DC, Lindahl-Jacobsen R, Rau R, Christensen K, Oksuzyan A (2018). Sex differences in the 1-year risk of dying following all-cause and cause-specific hospital admission after age 50 in comparison with a general and non-hospitalised population: a register-based cohort study of the Danish population. BMJ Open.

[CR14] Höhn A, Gampe J, Lindahl-Jacobsen R, Christensen K, Oksuyzan A (2020). Do men avoid seeking medical advice? A register-based analysis of gender-specific changes in primary healthcare use after first hospitalisation at ages 60+ in Denmark. J Epidemiol Community Health.

[CR15] Horiuchi S, Wilmoth JR, Pletcher SD (2008). A decomposition method based on a model of continuous change. Demography.

[CR16] Hunt K, Adamson J, Hewitt C, Nazareth I (2011). Do women consult more than men? A review of gender and consultation for back pain and headache. J Health Services Res Policy.

[CR17] Jensen AB (2014). Temporal disease trajectories condensed from population-wide registry data covering 6.2 million patients. Nat Commun.

[CR18] Karampampa K, Drefahl S, Andersson T, Ahlbom A, Modig K (2013). Trends in age at first hospital admission in relation to trends in life expectancy in Swedish men and women above the age of 60. BMJ Open.

[CR19] Lahti RA, Penttila A (2001). The validity of death certificates: routine validation of death certification and its effects on mortality statistics. Forensic Sci Int.

[CR20] Layes A, Asada Y, Kephart G (2012). Whiners and deniers–What does self-rated health measure?. Soc Sci Med.

[CR21] Lindahl-Jacobsen R, Rau R, Jeune B, Canudas-Romo V, Lenart A, Christensen K, Vaupel JW (2016). Rise, stagnation, and rise of Danish women’s life expectancy. Proc Natl Acad Sci USA.

[CR22] Luben R, Hayat S, Wareham N, Khaw KT (2016). Predicting admissions and time spent in hospital over a decade in a population-based record linkage study: the EPIC-Norfolk cohort. BMJ Open.

[CR23] Lynge E, Sandegaard JL, Rebolj M (2011). The Danish National Patient Register. Scand J Public Health.

[CR24] Modig K, Berglund A, Talbäck M, Ljung R, Ahlbom A (2017). Estimating incidence and prevalence from population registers: example from myocardial infarction. Scandinavian J Public Health.

[CR25] Modig K, Talbäck M, Ziegler L, Ahlbom A (2019). Temporal trends in incidence, recurrence and prevalence of stroke in an era of ageing populations, a longitudinal study of the total Swedish population. BMC Geriatr.

[CR26] Modig K, Rau R, Ahlbom A (2020). Life expectancy: what does it measure?. BMJ Open.

[CR27] Munk-Jørgensen P, Bertelsen A, Dahl AA, Lehtinen K, Lindström E, Tomasson K (1999). Implementation of ICD-10 in the Nordic countries Nordic. J Psychiatry.

[CR28] Oksuzyan A, Dańko MJ, Caputo J, Jasilionis D, Shkolnikov VM (2019). Is the story about sensitive women and stoical men true? Gender differences in health after adjustment for reporting behaviour. Soc Sci Med.

[CR29] Olejaz M, Juul AN, Rudkjøbing A, Okkels HB, Krasnik A, Hernández-Quevedo C (2012) Denmark health system review. Health Syst Transition 14:i–xxii, 1–192 22575801

[CR30] O'Malley KJ, Cook KF, Price MD, Wildes KR, Hurdle JF, Ashton CM (2005). Measuring diagnoses: ICD Code Accuracy. Health Serv Res.

[CR31] Pedersen KM, Andersen JS, Søndergaard J (2012). General Practice and Primary Health Care in Denmark The. J Am Board Fam Med.

[CR32] Ponnapalli KM (2005). A comparison of different methods for decomposition of changes in expectation of life at birth and differentials in life expectancy at birth. Demogr Res.

[CR33] Preston SH, Heuveline P, Guillot M (2001). Demography : measuring and modeling population processes.

[CR34] Riffe T (2018) DemoDecomp: decompose demographic functions.

[CR35] Schmidt M, Jacobsen JB, Lash TL, Bøtker HE, Sørensen HT (2012). 25 year trends in first time hospitalisation for acute myocardial infarction, subsequent short and long term mortality, and the prognostic impact of sex and comorbidity: a Danish nationwide cohort study. BMJ.

[CR36] Schmidt M, Pedersen L, Sørensen HT (2014). The Danish Civil Registration System as a tool in epidemiology. Eur J Epidemiol.

[CR37] Schmidt M, Schmidt SAJ, Sandegaard JL, Ehrenstein V, Pedersen L, Sørensen HT (2015). The Danish National Patient Registry: a review of content, data quality, and research potential. Clin Epidemiol.

[CR38] Seaman R, Höhn A, Lindahl-Jacobsen R, Martikainen P, van Raalte A, Christensen K (2020). Rethinking morbidity compression. Eur J Epidemiol.

[CR39] Simmonds SJ, Syddall HE, Walsh B, Evandrou M, Dennison EM, Cooper C, Aihie Sayer A (2014). Understanding NHS hospital admissions in England: linkage of Hospital episode statistics to the Hertfordshire cohort study. Age Ageing.

[CR40] Sundberg L, Agahi N, Fritzell J, Fors S (2018). Why is the gender gap in life expectancy decreasing? The impact of age- and cause-specific mortality in Sweden 1997–2014. Int J Public Health.

[CR41] Thorslund M, Wastesson JW, Agahi N, Lagergren M, Parker MG (2013). Eur J Ageing.

[CR42] Thun M, Peto R, Boreham J, Lopez AD (2012). Stages of the cigarette epidemic on entering its second century. Tob Control.

[CR43] Thygesen LC, Ersbøll AK (2014). When the entire population is the sample: strengths and limitations in register-based epidemiology. Eur J Epidemiol.

[CR44] Wang Y, Hunt K, Nazareth I, Freemantle N, Petersen I (2013). Do men consult less than women? An analysis of routinely collected UK general practice data. BMJ Open.

[CR45] Westergaard D, Moseley P, Sørup FKH, Baldi P, Brunak S (2019). Population-wide analysis of differences in disease progression patterns in men and women. Nat Commun.

