# Supplementary material for: Gender differences in time to first hospital admission at age 60 in Denmark, 1995–2014
Source: Eur J Ageing. 2021 Mar 27;18(4):443–51. doi: 10.1007/s10433-021-00614-w (PMC8563932; doi:10.1007/s10433-021-00614-w)
Supplement: Supplementary file 1 — Supplementary file1 (DOCX 12 kb) [file 10433_2021_614_MOESM1_ESM.docx]

**Supplementary Material 1: ICD-10 Coding**

| **Cause of admission category** | **ICD Codes** |
| --- | --- |
| Circulatory | I00-I099 |
| Respiratory | J00-J99 |
| Neoplasms | C00-D48*  *excluding C50, C51-C58, C60-C63 |
| Digestive | K00-K93 |
| Injury | S00-T98, V01-Y98 |
| Musculoskeletal | M00-M99 |
| Sex-specific | C50, C51-C58, C60-C63, N40-N51, N60-N64, N70-N77, N80-N98, O00-O99, Z30-39 |
| All other | All other, including missing and ill defined |

**Supplementary Table 1** ICD-10 codes assigned to each cause of admission category
