# Supplementary material for: Gender differences in time to first hospital admission at age 60 in Denmark, 1995–2014
Source: Eur J Ageing. 2021 Mar 27;18(4):443–51. doi: 10.1007/s10433-021-00614-w (PMC8563932; doi:10.1007/s10433-021-00614-w)
Supplement: Supplementary file 2 — Supplementary file2 (DOCX 73 kb) [file 10433_2021_614_MOESM2_ESM.docx]

## Supplementary Material 2: Sex-Specific Causes of Admission

We chose to identify sex-specific causes of admission as an independent cause of admission category. This was an attempt to make the other major categories (e.g. neoplasms) reflect causes of admission that both, men and women, could both be admitted to hospital for. The sex-specific causes of admission category grouped together eight diagnostic codes that were not constrained to one gender. It is common practice in health research to group together detailed diagnostic codes in order to decrease the potential for misclassification error (Lahti and Penttilä, 2001).

Supplementary figure 1 shows the admission rates for the detailed diagnostic codes that were grouped together into the sex-specific causes of admission category. Over the study period, the detailed diagnostic codes were generally following a decreasing trend with the exception of cancers of the male genital organs which increased sharply between 2005 and 2009. When looking at the detailed diagnostic codes for sex specific causes it is plausible that the sharp increases and sharp decreases over a short period of time (e.g. Genitourinary System: Male Genital Organs, Cancers: Breast, Cancers: Male Genital Organs) are a sign that admission or treatment strategies changed.


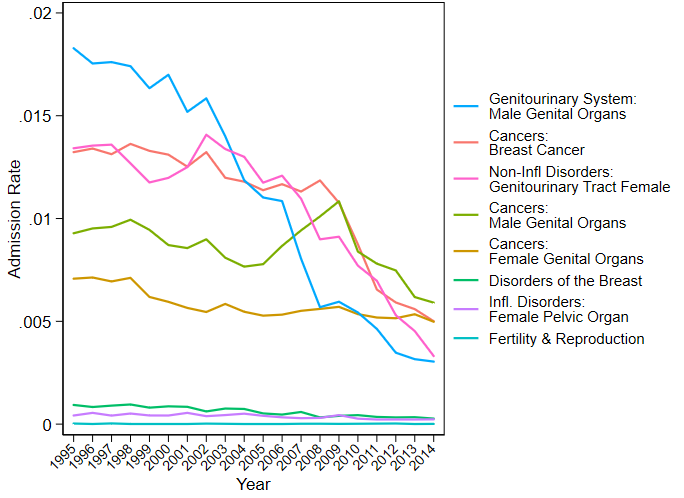


**Supplementary Fig 1** Detailed incidence rates for causes of admissions categorised as sex-specific causes of admission, 1995 to 2014
