# Supplementary material for: Gender differences in time to first hospital admission at age 60 in Denmark, 1995–2014
Source: Eur J Ageing. 2021 Mar 27;18(4):443–51. doi: 10.1007/s10433-021-00614-w (PMC8563932; doi:10.1007/s10433-021-00614-w)
Supplement: Supplementary file 3 — Supplementary file3 (DOCX 52 kb) [file 10433_2021_614_MOESM3_ESM.docx]

## Supplementary Material 3: Sensitivity Analysis

When analysing inpatient admission, the data provides an “in date” and “out date” variable, and a separate variable for the “number of treatments days”. The length of time captured between the “in date” and “out date” variable usually corresponds to the length of time in the “number of treatment days”. When analysing outpatient admissions, the data provide the same variables. However, the length of time estimated from the “in date” and “out date” variable rarely corresponds with the “number of treatment days”. This is because inpatient treatment days are consecutive days while outpatient treatment days are not consecutive and can take place intermittently throughout an extended period of time. For example, three treatment days for an outpatient admission could occur over a period of six months. Therefore, the sensitivity results should be interpreted with caution as the length of treatment is not directly comparable between inpatient and outpatient admissions.

Supplementary figure 2 shows the trends in remaining years to admission when including outpatient and emergency admissions. Supplementary figure 3 shows that the magnitude of gender differences in the time to hospital admission at age 60, when including all types of hospital admissions, was small and slightly decreased over the study period.


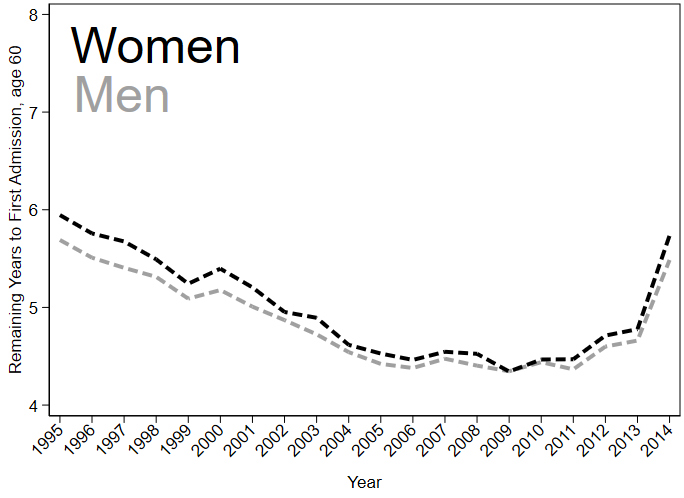


**Supplementary Fig 2** Trends in remaining years to first hospital admission for Danish men and women aged 60, including inpatient, outpatient and emergency admissions with a minimum of two treatment days, 1995 to 2014.
